# Bilateral Superolateral Fracture Dislocation of the Temporomandibular Joint (TMJ) Following Trauma: A Report of a Unique Case

**DOI:** 10.7759/cureus.108237

**Published:** 2026-05-04

**Authors:** Ganesh Parth Gorasia, Satnam Rehal, Waleed Khan, Rory O'Connor

**Affiliations:** 1 Maxillofacial Surgery, Queen’s Medical Centre, Nottingham, GBR; 2 Maxillofacial Surgery, Queen's Medical Centre, Nottingham, GBR; 3 Maxillofacial Surgery, Leicester Royal Infirmary, Leicester, GBR

**Keywords:** 3d-planning, facial trauma surgery, intraoral scanning, mandibular condyle, temporomandibular joint

## Abstract

Traumatic temporomandibular joint (TMJ) dislocation is well described; however, superolateral displacement of the mandibular condyle relative to the zygomatic arch is rare, particularly when occurring bilaterally in the setting of panfacial trauma.

A 17-year-old male sustained high-energy trauma in a road traffic collision and was intubated at the scene (Glasgow Coma Scale (GCS) score of 5). Trauma CT demonstrated multisystem injuries and complex maxillofacial fractures, including Le Fort II fractures, comminuted symphyseal mandibular fracture, bilateral high condylar head fractures, and bilateral superolateral TMJ fracture-dislocation. Conventional dental impressions were not feasible due to the endotracheal tube; a digital intraoral scan (3Shape TRIOS) was used to capture occlusion for 3D planning and fabrication of patient-specific occlusal splints. Following medical stabilization, the patient underwent submental intubation, open reduction of both TMJs via preauricular approaches, and open reduction and internal fixation of midface and mandibular fractures.

Bilateral superolateral TMJ fracture-dislocation can present a mechanical block to closed reduction. Digital occlusal capture combined with CT-based 3D planning may assist in restoring mandibular width and achieving accurate maxillomandibular reduction in complex panfacial trauma.

## Introduction

Traumatic temporomandibular joint (TMJ) dislocation most commonly occurs in the anterior direction [1,2]. In high-energy mechanisms, less common displacement patterns may occur and can be difficult to recognize on initial assessment, particularly when there is extensive associated facial injury and soft-tissue swelling [1,2]. In this report, superolateral displacement refers to the displacement of the mandibular condylar segment upwards (superiorly) and outwards (laterally) relative to the glenoid fossa and zygomatic arch, which may create a mechanical obstruction to closed reduction. Panfacial trauma describes injury involving multiple regions of the facial skeleton (typically the upper, middle, and lower facial thirds), disrupting bony buttresses and compromising mandibular stability and occlusion.

Restoring occlusion is a key objective in the management of complex facial fractures because it provides a reference for accurate reduction and fixation of the midface and mandible. However, obtaining an accurate dental record can be challenging when access is limited by airway devices, restricted mouth opening, swelling, or unstable fractures. Digital intraoral scanning enables rapid capture of the dentition without conventional impressions and supports digital occlusal planning. This approach allows the dental model to be shared and reviewed easily, merged with CT-derived 3D reconstructions, and used to fabricate patient-specific occlusal splints. In turn, splint-guided maxillomandibular reduction can help re-establish the planned bite and transverse mandibular width intraoperatively, reducing reliance on visual estimation and improving reproducibility during fixation [3].

We present this case to (1) raise awareness of this uncommon displacement pattern, which may be missed on initial assessment, and (2) describe a practical workflow using intraoral scanning and 3D planning to restore occlusion and mandibular width during operative management of complex facial fractures.

## Case presentation

**Figure 1:**
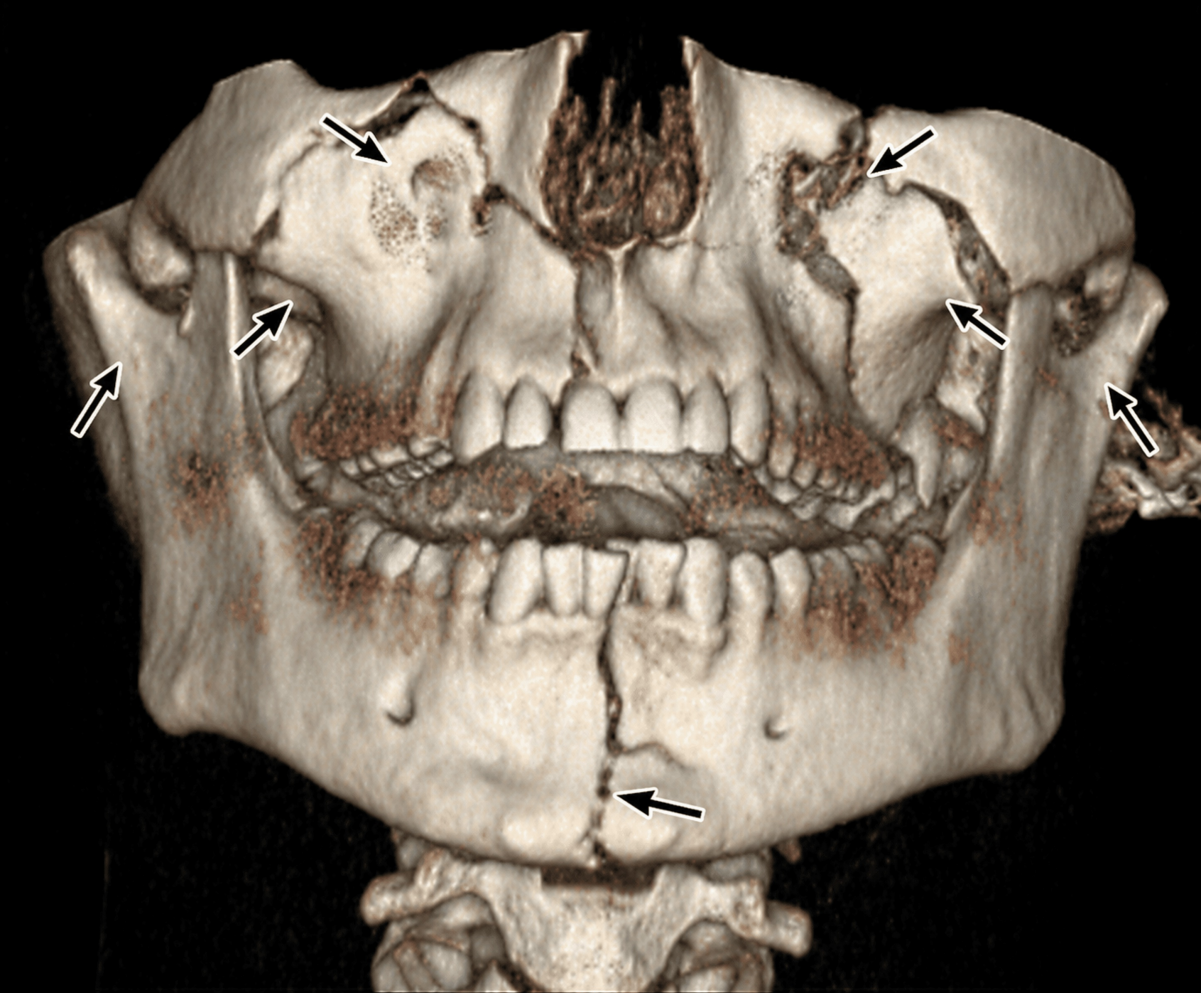
3D reconstruction of CT head and facial bones demonstrating a mandibular symphyseal fracture with a comminuted Le Fort II fracture pattern, as well as bilateral superolateral TMJ dislocation. The lateral arrows indicate the bilaterally displaced condylar fragments consistent with superolateral TMJ dislocation; the upper and midfacial arrows indicate the Le Fort II fracture lines; and the inferior arrow indicates the mandibular symphyseal fracture line. CT, computed tomography; TMJ, temporomandibular joint

**Figure 2:**
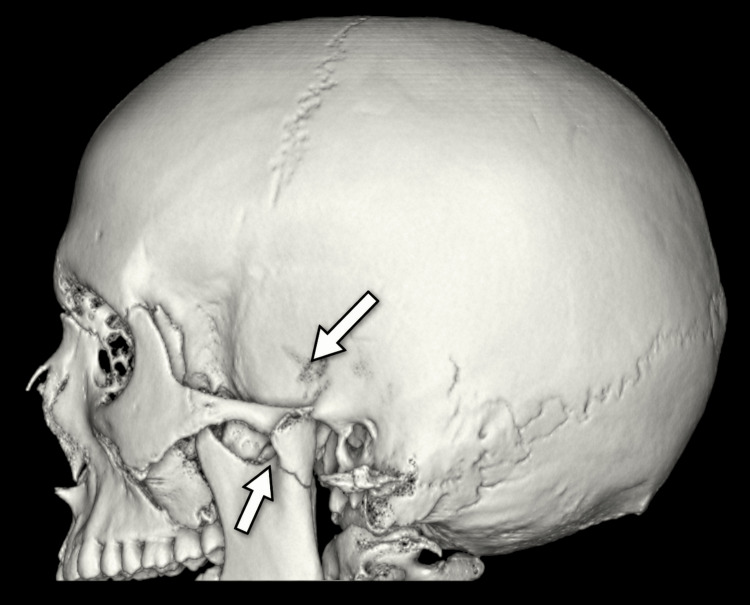
Preoperative 3D CT lateral volume-rendered image demonstrating a left-sided condylar fracture with superolateral TMJ fracture-dislocation. The upper arrow indicates the superolaterally displaced condylar fragment relative to the zygomatic arch, while the lower arrow indicates the fracture line at the condylar head/neck region. CT, computed tomography; TMJ, temporomandibular joint

**Figure 3:**
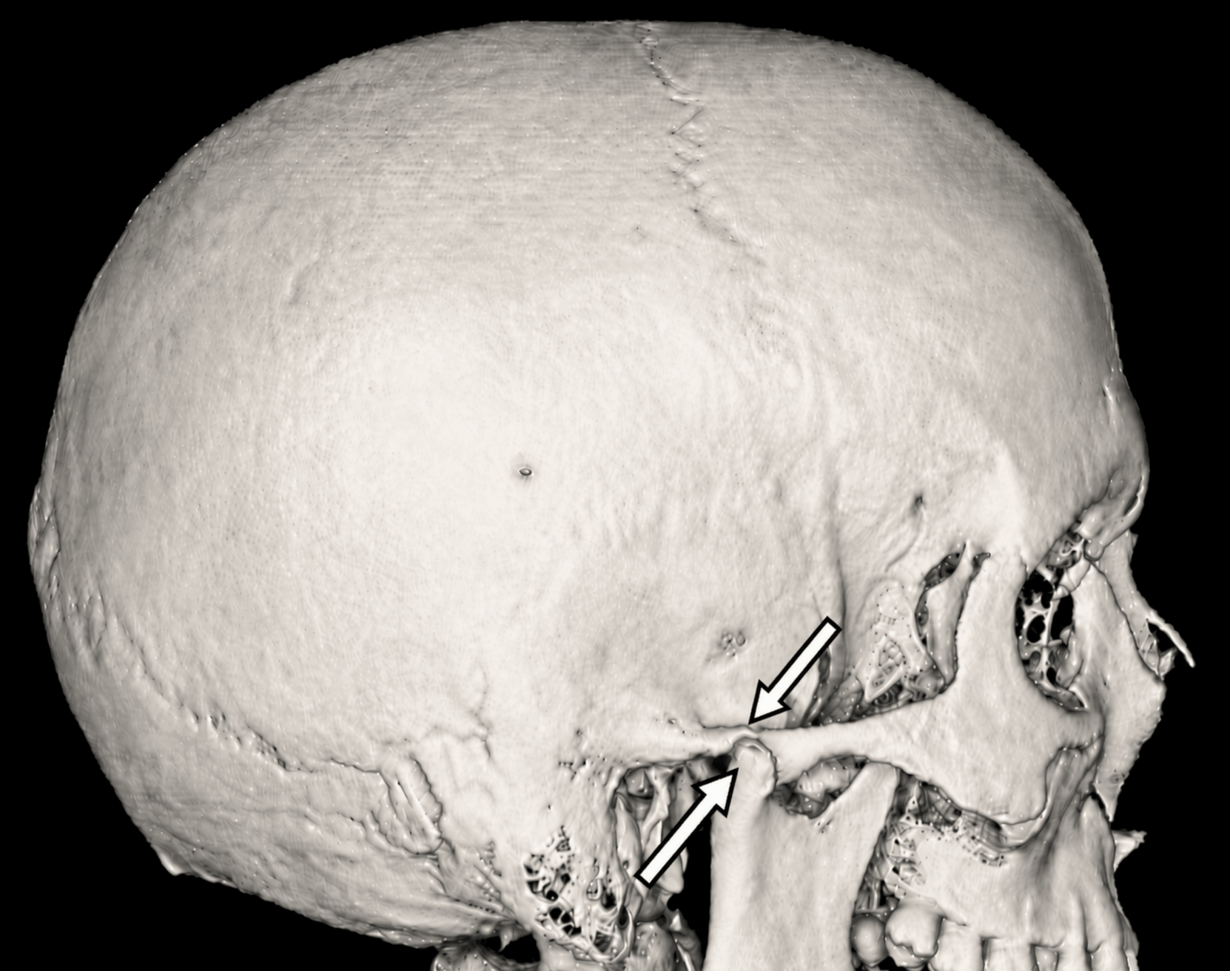
3D CT lateral volume-rendered image demonstrating a condylar fracture with superolateral displacement of the right TMJ relative to the zygomatic arch. The upper arrow indicates the superolaterally displaced right condylar fragment, and the lower arrow indicates the fracture line through the condylar head/neck region. CT, computed tomography; TMJ, temporomandibular joint

In May 2024, a previously well 17-year-old male motorcyclist was involved in a high-speed road traffic collision. At the scene, he had a Glasgow Coma Scale (GCS) score of 5 and was intubated. He was transferred to the regional Major Trauma Centre at Queen’s Medical Centre. Trauma CT demonstrated intracranial and multisystem injuries, including a subdural hematoma, splenic lacerations, sternal and metacarpal fractures, and pulmonary contusions. Maxillofacial CT demonstrated Le Fort II fractures, a comminuted mandibular symphyseal fracture, bilateral high condylar head fractures, and bilateral superolateral TMJ fracture-dislocation with the condylar segments displaced lateral/superior to the zygomatic arches (Figures 1-3).

**Figure 4:**
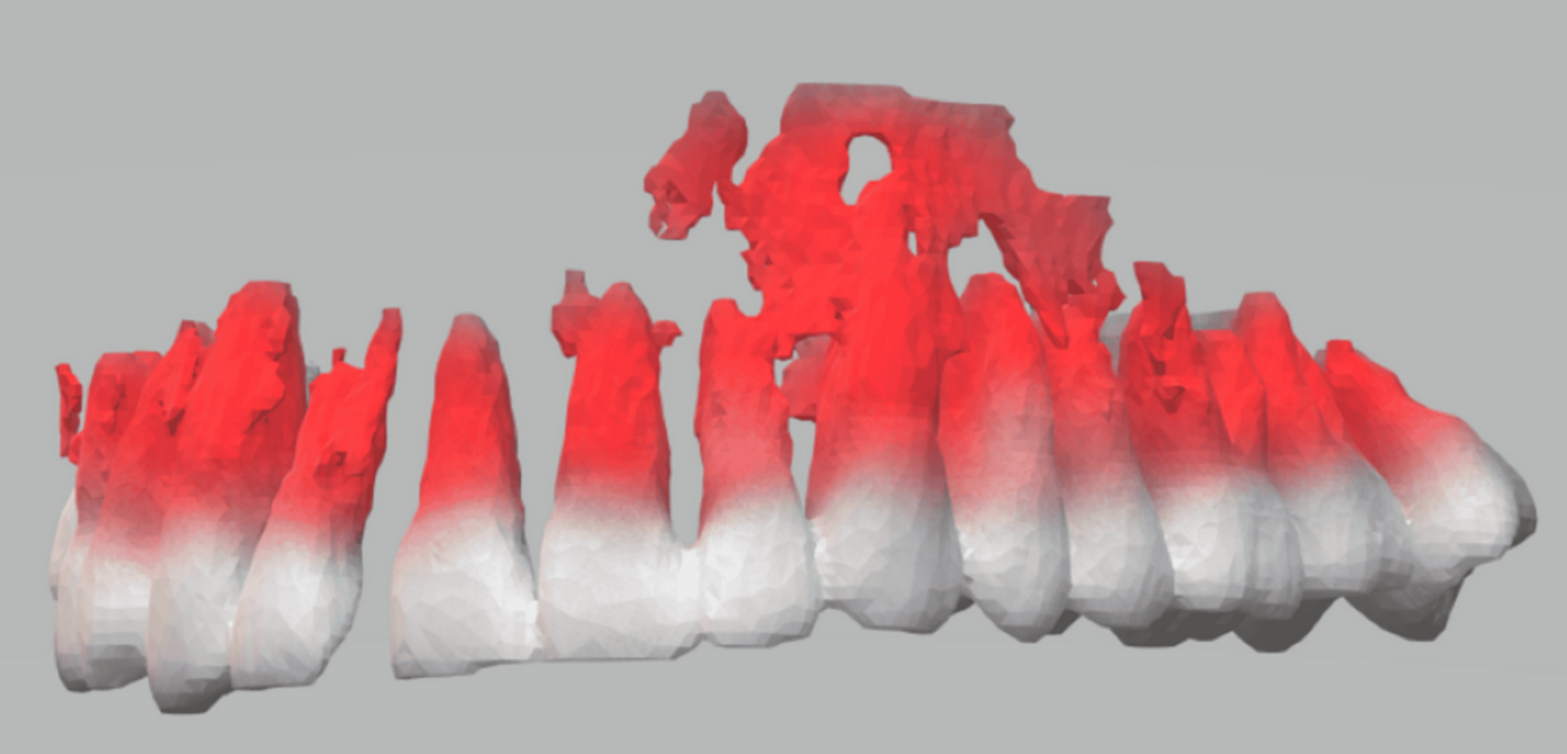
Digital intraoral scan obtained with a 3Shape TRIOS (3Shape, Copenhagen, Denmark) scanner for occlusal reconstruction and 3D planning. Surface-rendered intraoral scan of the maxillary dentition showing the teeth (white) and gingival/soft-tissue surfaces (red). The scan was used to generate a digital dental model for preoperative occlusal registration and integration with CT-based 3D modeling when conventional impressions were not feasible. CT, computed tomography

**Figure 5:**
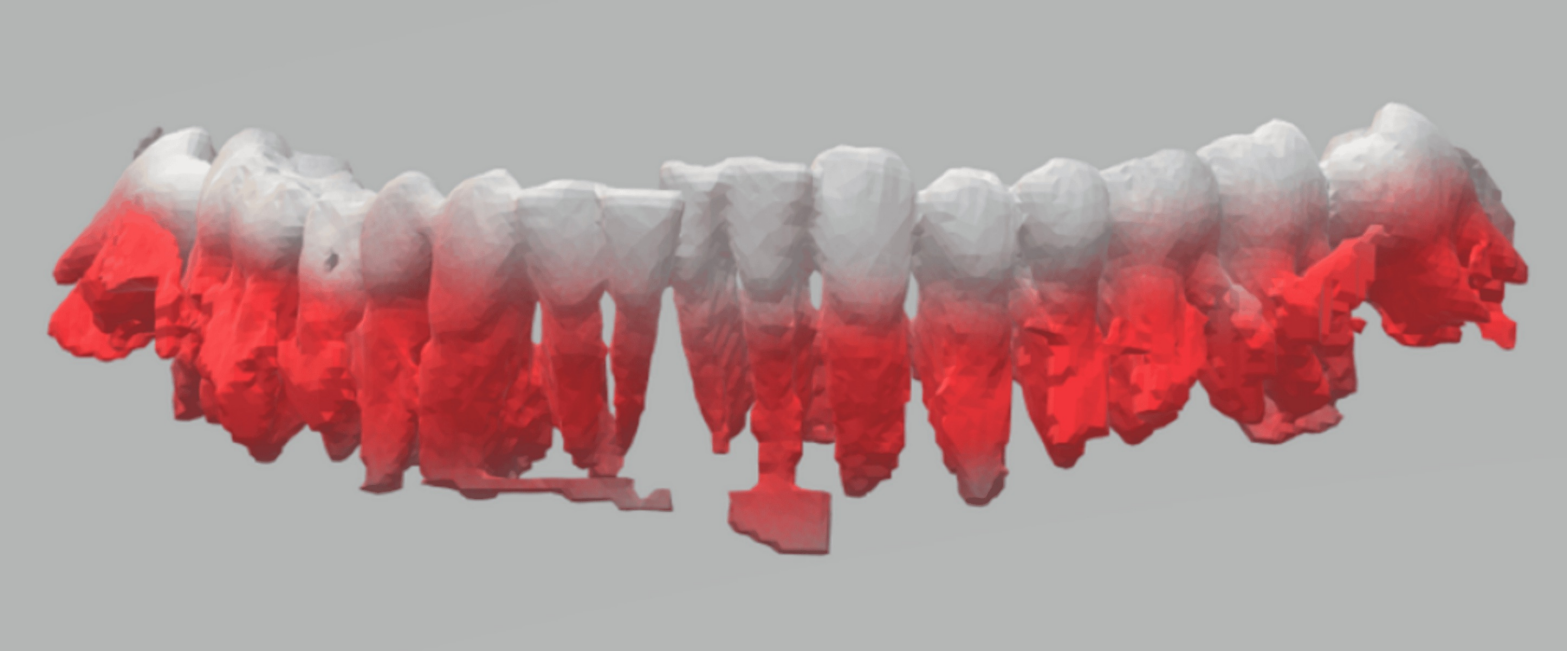
Digital intraoral scan of the mandibular dentition acquired with a 3Shape TRIOS scanner (3Shape, Copenhagen, Denmark). Surface-rendered TRIOS scan demonstrating the mandibular teeth (white) and gingival/soft-tissue surfaces (red). This digital dental model was used to provide an accurate occlusal record for virtual surgical planning and fabrication of patient-specific occlusal splints when conventional impressions were not feasible.

The patient was managed in the Intensive Therapy Unit for head injury and physiological stabilization. Pre-operative occlusal registration was required for surgical planning; however, conventional impressions were not feasible due to the endotracheal tube. A digital intraoral scan was obtained using a 3Shape TRIOS (3Shape, Copenhagen, Denmark) intraoral scanner to capture the dentition and occlusal relationship, which was combined with CT-derived 3D models to support planning and fabrication of custom occlusal splints (Figures 4-5). Digital occlusal planning offers several advantages in complex facial trauma: it provides a rapid, accurate record of the dentition when conventional impressions are impractical (e.g., due to intubation, limited mouth opening, or unstable fractures), and it supports virtual planning and fabrication of patient-specific splints to reproduce occlusion and mandibular width. This can reduce intraoperative guesswork, facilitate communication within the multidisciplinary team, and help achieve predictable maxillomandibular reduction.

**Figure 6:**
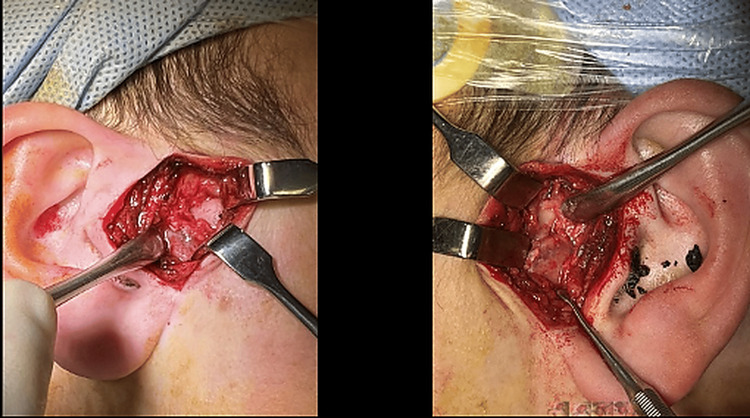
Bilateral preauricular open reduction of TMJ fracture-dislocations. Left and right intraoperative views showing preauricular exposure and instrument-assisted reduction of the displaced condylar fragments under direct vision. TMJ, temporomandibular joint

Definitive maxillofacial fixation was deferred until the patient was physiologically stable and safe for prolonged surgery given the associated intracranial and multisystem injuries. Once stable, the patient underwent temporary submental intubation (provide unobstructed intraoral access for maxillomandibular fixation and occlusal splint placement) and definitive operative management including open reduction of both TMJs via bilateral preauricular approaches and open reduction/internal fixation of the midface and mandibular fractures. Given the comminuted symphyseal fracture and loss of transverse mandibular stability, occlusal control was established intraoperatively using a custom occlusal splint and maxillomandibular fixation to reproduce the planned mandibular width and occlusion. The TMJs were exposed through bilateral preauricular incisions to confirm superolateral displacement of the condylar fragments relative to the zygomatic arch. Reduction was performed sequentially, one side at a time: under direct vision the condylar fragment was guided inferiorly and medially to clear the zygomatic arch and then seated into the glenoid fossa. Mandibular positioning during reduction was controlled through the dentition/MMF and splint (rather than traction on the mandibular body) to avoid further displacement at the symphysis. Following reduction, stability was assessed and the procedure proceeded to definitive fixation of the mandibular symphysis and midface fractures with occlusal verification at the end of the case. Intraoperative images are shown in Figure 6.

**Figure 7:**
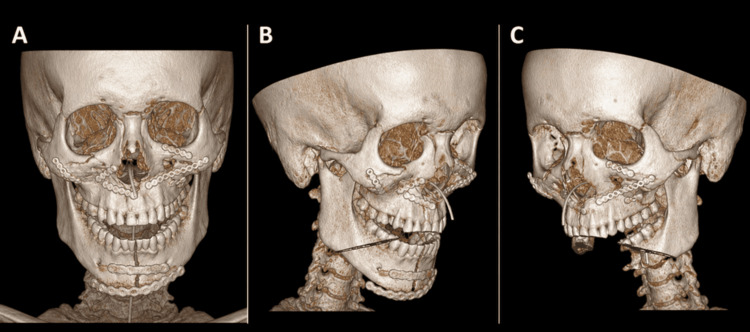
Postoperative volume-rendered CT images show (A) a frontal view, (B) a left oblique/lateral view, and (C) a right oblique/lateral view. The images demonstrate stable fixation of the midface and mandible, with maintained alignment and reduction of both mandibular condyles following operative management. CT, computed tomography

Postoperatively, the patient was managed in line with his associated injuries and was reviewed by the maxillofacial team during inpatient recovery. Occlusion was assessed clinically and compared with the planned occlusal relationship, demonstrating stable intercuspation without open bite. TMJ examination demonstrated no recurrent dislocation and improving mandibular range of motion with physiotherapy. Postoperative CT confirmed maintenance of reduction of both condyles within the glenoid fossae and stable fixation (Figures 7A-7C). There was no evidence of post operative complications. The patient was followed up for 12 months, during which time he maintained stable occlusion and TMJ function with no late complications (including no recurrent dislocation, malocclusion, infection, or hardware-related problems).

## Discussion

TMJ dislocations can be categorized as acute, recurrent (habitual), or chronic, with the latter defined as dislocations left untreated or inadequately treated for 72 hours or more [4]. Acute factors include trauma, iatrogenic causes (including intubation and prolonged dental or ENT procedures), and spontaneous events (such as yawning and emesis). Recurrent and chronic cases can be attributed to unfavorable anatomy and systemic diseases, such as Ehlers-Danlos syndrome, Huntington’s disease, and muscular dystrophy [1,2]. Superior displacement of the mandibular condyle into the middle cranial fossa is rare and has been described in the literature [5]. In contrast, superolateral displacement of the mandibular condyle relative to the zygomatic arch is uncommon and may present a mechanical obstruction to closed reduction, particularly in high-energy trauma.

In 460 BC, Hippocrates first described his conventional manual method of reducing anterior TMJ dislocations. The clinician stands in front of the patient with thumbs placed lateral to the mandibular molars; force is then applied in an inferior and posterior direction to manipulate the mandibular condyles back into the glenoid fossa. This technique was later modified by Lewis in 1981, who advocated placing the fingers on the occlusal surfaces of the mandibular molars to apply a downward force while simultaneously elevating the chin and transposing the entire mandible posteriorly [6].

Given the significant mechanism of injury in our case, a Guardsman-type fracture pattern might have been anticipated; however, the mandibular condyles were displaced superolaterally in relation to the zygomatic arch [7]. The condylar position created a mechanical obstruction, making conventional closed reduction techniques unfavorable. As a result, an open surgical approach was implemented. Bilateral preauricular incisions were made to expose and confirm the position of the condyles, which were then guided beneath the zygomatic arch and reduced back into their anatomical position within the glenoid fossa sequentially, one side at a time. The lateral joint capsule was sutured to improve stability.

It is important to note that this fracture-dislocation pattern resulted in loss of control of mandibular position in all three planes of space, particularly causing transverse widening of the mandible. Accurate maxillomandibular reduction could only be achieved with custom-made 3D splints that fitted closely to the teeth. Therefore, the combination of an intraoral scan with 3D modeling of the patient's CT scan was invaluable in reproducing occlusion and restoring mandibular width [3].

## Conclusions

Bilateral superolateral TMJ fracture-dislocation is a rare injury pattern that may preclude closed reduction due to mechanical obstruction. In complex panfacial trauma, where mandibular width and occlusion are unstable, digital intraoral scanning combined with CT-based 3D planning can assist in occlusal reconstruction and operative reduction. In this case, the patient returned to normal function following rehabilitation for all injuries and, at follow-up, demonstrated satisfactory outcomes.
